# Postoperative Atrial Fibrillation

**DOI:** 10.5402/2011/203179

**Published:** 2011-05-22

**Authors:** C. Chelazzi, G. Villa, A. R. De Gaudio

**Affiliations:** Section of Anesthesiology and Intensive Care, Department of Critical Care, University of Florence, 50121 Florence, Italy

## Abstract

Postoperative atrial fibrillation (POAF) is common among surgical patients and associated with a worse outcome. Pathophysiology of POAF is not fully disclosed, and several perioperative factors could be involved. Direct cardiac stimulation from perioperative use of catecholamines or increased sympathetic outflow from volume loss/anaemia/pain may play a role. Metabolic alterations, such as hypo-/hyperglycaemia and electrolyte disturbances, may also contribute to POAF. Moreover, inflammation, both systemic and local, may play a role in its pathogenesis. Strategies to prevent POAF aim at reducing its incidence and ameliorate global outcome of surgical patients. Nonpharmacological prophylaxis includes an adequate control of postoperative pain, the use of thoracic epidural analgesia, optimization of perioperative oxygen delivery, and, possibly, modulation of surgery-associated inflammatory response with immunonutrition and antioxidants. Perioperative potassium and magnesium depletion should be corrected. The impact of those interventions on patients outcome needs to be further investigated.

## 1. Introduction

Postoperative atrial fibrillation (POAF) is common both after cardiothoracic and noncardiothoracic surgery [1]. In patients undergoing cardiothoracic surgery an incidence of 16–46% has been reported, depending on the extent of postoperative monitoring used and the specific surgical procedures [2–9]. In patients undergoing noncardiothoracic surgery, reported incidence of POAF varies between 0.4% and 12% [9, 10]. POAF can be observed during all the postoperative course, with a peak between the second and fifth postoperative day [11].

Risk of developing POAF may be related to several epidemiological and perioperative predictive factors. General factors include older age, male gender, obesity, preexisting congestive heart failure, chronic renal failure, or COPD which are all risk factors for POAF [12, 13]. In noncardiothoracic surgery, predictors for POAF are preexisting valvular disease and asthma, intra-abdominal and major vascular procedures, and intraoperative hypotension [14]. 

Even though POAF can be self-limiting, it may be associated with hemodynamic derangements, postoperative stroke, perioperative myocardial infarction, ventricular arrhythmias, and heart failure [15, 16]. In many reports, development of POAF is associated with a longer hospital stay, greater morbidity and mortality, and increased costs [11, 17]. In patients at risk of POAF, additional interventions, both pharmacological and nonpharmacological, are suggested in order to prevent its clinical consequences [11]. Amiodarone, beta-blockers, and magnesium supplementation are the mainstay of pharmacological prophylaxis in patients at risk [11]. Nonpharmacological prophylaxis may include several interventions during all the perioperative time. The aim of this study is to review nonpharmacological prophylaxis of POAF in light of its complex pathophysiology.

## 2. Pathophysiology and Nonpharmacologic Treatment

**Figure 1:**
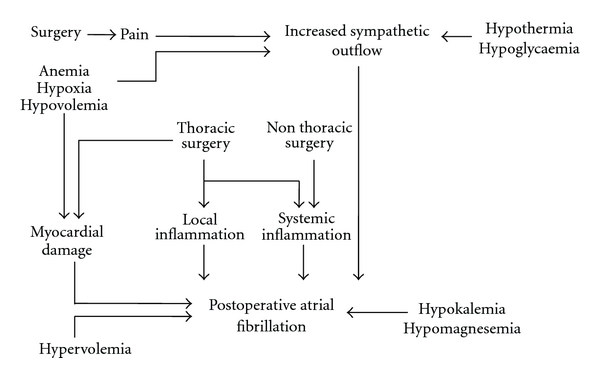
Pathophysiology of postoperative atrial fibrillation (see text for details).

The electrophysiological mechanisms involved in POAF are not fully disclosed. Classically, atrial fibrillation can be linked to alteration in atrial refractoriness, slowing of atrial conduction, and/or reentry in wavelets of excitation in the atria [18, 19]. It has been proposed that patients who develop POAF may already have an electrophysiological substrate for this arrhythmia before surgery [18]. Several perioperative factors would be able to trigger atrial fibrillation. In patient undergoing cardiothoracic surgery, the intraoperative trauma per se, manipulation of the heart, local inflammation with or without pericarditis, and elevation in atrial pressure from postoperative ventricular stunning may all predispose to alterations in refractoriness and/or local reentry [18]. In all surgical patients, atrial fibrillation can be also related to specific factors, such as direct cardiac stimulation from perioperative use of catecholamines or reflex sympathetic activation from volume loss/anaemia/pain, fever, and hypo-/hyperglycaemia [20–22]. There is also evidence that inflammation, both systemic and local, may play a role in POAF pathogenesis (see Figure 1) [23]. However, exact mechanisms of POAF after noncardiothoracic surgery are not fully understood [10].

### 2.1. Mechanical Factors, Pain, and Sympathetic Stimulation

Atrial fibrillation is commonly described after pulmonary or esophageal surgery [24–30]. During cardiothoracic surgery, mechanical manipulation of heart and pericardium leads to a local tissue trauma and local inflammatory response which may both lead to POAF [31–33]. Heart manipulation may be responsible also for a direct and local imbalance between sympathetic and parasympathetic tone, (see Figure 1). Direct injury to myocardial sympathetic nerve fibers may alter the autonomic modulation of atrial myocardial cells, increasing their sensitivity to catecholamines, thus contributing to POAF. Systemic sympathetic tone can be increased as well, due to a systemic inflammatory response to surgical trauma and pain, and circulating catecholamines may act on the sensitized atrial myocardium shortening refractory period, causing atrial reentry or promoting triggered automaticity to produce arrhythmias [32]. Thus, perioperative beta-blockade exerts similar effects and can play a role in limiting postoperative atrial arrhythmias [34].

Pain itself triggers a sympathetic response which contributes to postoperative arrhythmias (see Figure 1). Pain-induced sympathetic outflow and the imbalance between sympathetic and parasympathetic activity produce atrial ectopic beats and, in susceptible patients, trigger POAF (see Figure 1) [35]. There is experimental evidence that adequate postoperative pain relief is associated with a reduced incidence of POAF. In a prospective case-control study, Hooten and colleagues found a higher incidence of POAF in patients after thoracic surgery with poor control of postoperative pain [36].

**Figure 2:**
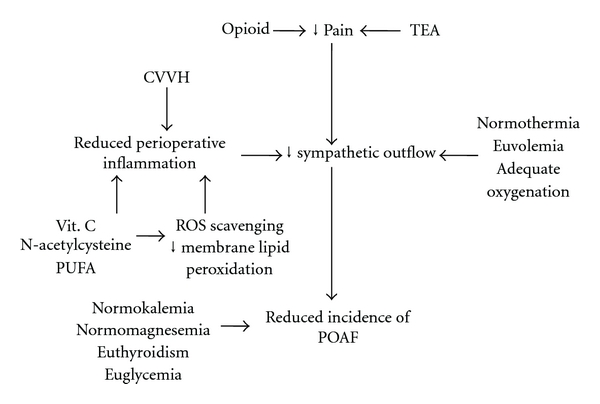
Nonpharmacological prophylaxis of postoperative atrial fibrillation (see text for details).

Thoracic epidural anesthesia (TEA) may reduce incidence of atrial arrhythmias (see Figure 2) [37]. Reasons include a direct negative chronotropic effect on heart, a better coronary blood flow and myocardial oxygenation, and reduced sympathetic outflow from perioperative pain (see Figure 2) [38]. In cardiothoracic surgery, postoperative TEA with local anesthetics, for example, bupivacaine, induces a variable degree of sympathetic blockade, which can be responsible for the observed reduction in postoperative cardiac tachyarrhythmias [32]. There is also a general agreement on the fact that TEA allows a better control on postoperative pain, thus reducing the release of catecholamines (see Figure 2) [32, 39, 40]. Moreover, TEA can reduce myocardial sensitivity to circulating catecholamines [41]. A TEA-mediated increase of myocardial vagal tone can also be partly responsible for its chronotropic effects (see Figure 2) [38]. Simeoforidou and colleagues measured heart rate variability in patients who underwent thoracotomy and showed a decreased cardiac sympathetic outflow in those patients on TEA compared to those who received patient-controlled i.v. morphine [42]. Intraoperative TEA can prevent POAF as well. Scott and colleagues reported a reduced incidence of postoperative atrial arrhythmias in patients who undergone a combined general-epidural anesthesia as compared to those who received general anesthesia alone (10.2% versus 22.3%) [37].

Interestingly, some authors state that atrial arrhythmias could be linked to a postoperative loss of parasympathetic state, which exerts inhibitory effect on atrial myocardium, and not to an enhanced sympathetic tone. In a recent, small trial, Jiang and colleagues found that, after pulmonary resection, a patient-controlled infusion of opioids (fentanyl and tramadol) can reduce postoperative supraventricular arrhythmias more than patient-controlled epidural analgesia with ropivacaine [35]. They argue that restoring parasympathetic stimulation on atrial myocardium, through opioid infusion, might be responsible for this result. However, actual evidence is that epidural analgesia is superior to systemic opioid in terms of outcome for patients undergoing cardiothoracic and major abdominal procedures, including abdominal vascular surgery and colonic cancer resection [43].

### 2.2. Inflammation and Anti-Inflammatory Strategies

During surgical trauma, a systemic inflammatory response is evident, and its intensity mirrors the degree of surgical stress [44]. Takenaka et al. demonstrated that serum levels of inflammatory markers, such as interleukin-6 (IL-6) and C-reactive protein (CRP), correlated with clinical determinants of surgical stress, such as duration of surgery, length of surgical wound, or intensity of pain [45]. 

There is mounting evidence showing the influence of systemic inflammation in the pathogenesis of atrial fibrillation (see Figure 1). Circulating levels of proinflammatory cytokines, like IL-6 and TNF-alpha, are elevated in patients with atrial arrhythmias [23]. Higher levels of complement, CRP or white blood cells, can be found in patients who develop POAF when compared to controls [46]. Considering patients undergoing elective cardiac surgery, Lamm and colleagues demonstrated that a more pronounced increase in postoperative markers of inflammation independently predicts development of POAF [47]. Furthermore, local myocardial inflammation may contribute to pathogenesis of POAF (see Figure 1) [48].

Both systemic and local inflammation may foster an oxidative injury with release of reactive oxygen species (ROS). ROS can induce an electrical myocardial remodelling, characterized by a diminished effective refractory period to action potential, thus precipitating POAF (see Figure 2) [49]. Indeed, there are experimental evidences that a oxidative injury occurs in myocardial tissues of patients with atrial fibrillation [50], and many studies have shown increased levels of serum myocardial oxidation markers, such as peroxynitrite and superoxide, in fibrillating patients after surgery [50–52]. NAD(P)H oxidase, the main source of superoxide in the atria, was found at higher levels in POAF-developing patients than in those who do not develop this arrhythmia [53]. Based on these experimental observations, perioperative supplementation of antioxidant could contribute in reducing POAF (see Figure 2). 

Antioxidants, including vitamin C, N-acetylcysteine, statins, are an heterogeneous group of molecules which have proved to decrease serum levels of molecular markers of cellular oxidative stress in patients undergoing cardiac surgery. They also can reduce the incidence of POAF (see Figure 2) [54, 55]. Furthermore it has been hypothesized that one of the mechanisms by which classic anti-AF drugs act is related with the ability to ROS scavenging and protection against membrane lipid peroxidation [56]. Numerous lines of evidence show that a reinforcement of the antioxidant defence system diminishes the vulnerability of myocardium to the effect of increased ROS. In support of this view, prevention of POAF by classic antioxidants such as N-acetylcysteine and statins has been reported [57]. In a prospective case-control study, Carnet and colleagues tested the effects of perioperative vitamin C supplementation in POAF incidence on 43 patients undergoing cardiac surgery. Incidence of POAF in the treated group was 16% versus 35% of the control group [58]. There is also evidence that oral administration of ascorbic acid in the perioperative period would be able to decrease POAF incidence about 50% [58]. Despite these lines of evidence, perioperative supplementation of antioxidants is not routinely undertaken [59].

Modulating the perioperative inflammatory response through the administration of immunonutrients could potentially reduce postoperative complications, including POAF (see Figure 2) [60]. Polyunsaturated omega-3 fatty acids (PUFAs) have been suggested to have direct effects on cardiac microsomal calcium/magnesium adenosine triphosphatase and voltage-gated sodium channels [61]. Clinical studies have shown that PUFAs reduce the incidence of atrial fibrillation as well as ventricular arrhythmias in patients with myocardial infarction [62] and implanted defibrillators [63]. In a prospective randomized prospective survey, Calò et al. [64]. randomized 160 patients undergoing CABG to receive either PUFA 2 g/day for at least 5 days postoperatively. The authors demonstrated that PUFAs were able to reduce the incidence of POAF by 65%. This effect was associated with a significant reduction in hospital length of stay (*P* = .017). 

Renal continuous venovenous hemofiltration (CVVH) and blood depuration techniques are increasingly employed to reduce or modulate the systemic inflammatory response in critically ill patients [65]. CVVH has been employed to reduce the inflammatory response associated with cardiothoracic surgery [66]. Indeed, in patients undergoing postoperative renal replacement therapy, hemofiltration effluent has been shown to contain tumor necrosis factor-alpha, interleukin-6, C3a, and C5a complexes [66–68]. Intraoperative CVVH during cardio-pulmonary bypass (CPB) has been shown to remove low-molecular-weight molecules from the plasma, including inflammatory mediators like cytokines [66, 67]. Due to the role of inflammation in the pathogenesis of POAF, it has been hypothesized that CVVH during CPB might decrease the incidence of AF after cardiac surgery [69]. However, timing of perioperative CVVH is still a matter of debate. AF typically occurs 48–96 h after surgery, and a short period of attenuating the inflammatory cascade during the operation probably has little benefit in preventing more delayed complications. In fact, it has been shown that C-reactive protein levels do not peak until postoperative day 2, and complement C3b/c levels undergo a secondary elevation between postoperative days 2 and 4 [46, 70]. This late peak in circulating levels of inflammatory mediators likely further negates the effects of a therapy confined to the intraoperative period [69]. Furthermore, for patients undergoing noncardiac surgery, routine application of CVVH is not indicated.

### 2.3. Volemia

Development of POAF can be related to hypovolemia and hypotension (see Figure 1) [71]. Blood and/or fluid loss decrease venous return to right atrium, thus reducing stroke volume and cardiac output. The same alterations can be the consequence of anesthesia-induced cardiovascular depression. The decreased tissue oxygen delivery stimulates the release of endogenous catecholamines. In hypovolemic patients, hemodynamic variations are finalized to maintain an adequate tissue perfusion and oxygenation, through an increase in heart rate, peripheral vascular resistance, and venomotor tone. These effects aim to restore venous return toward the heart and to maintain an adequate stroke volume. Secretion of catecholamines by the sympathetic system is crucial. In the surgical patient, loss of fluids and hemorrhage can lead to reflex sympathetic hyperactivity, which may have proarrhythmic on heart [72]. Noradrenaline, dopamine, or dobutamine infusion may play a role as well. Thus, in postoperative patients, new-onset POAF should prompt a differential diagnosis to rule out surgical bleeding.

Intraoperative hypovolemia, together with hypoxia and anemia, may lead to a relative ischemia of atrial cells and myocardial conduction tissue, altering the cell electric properties and leading to arrhythmias, both ventricular and supraventricular [26, 73, 74]. 

Hypervolemia is related to POAF through a mechanism of mechanical stimulation of the right atrium: the “mechanical-electrical feedback” [71, 75–78]. It has been shown that onset and duration of atrial cells' membrane action potential are affected by changes in the length of the ventricular cells [78–80]. Thereafter, myocardial stretch, in both isolated and in situ hearts, has been shown to induce ventricular arrhythmias because of the occurrence of transient depolarizations and shortening of the refractory period [81]. The large volume of fluid infused during perioperative period, especially in general and vascular surgery, could increase atrium diastolic volume and reversibly reduce compliance, altering electrical properties of atrial cells [82]. Consequent increased excitability may trigger POAF [83, 84].

Indeed, the Atrial Fibrillation Suppression Trial II showed that patients who developed POAF after cardiothoracic surgery received 1 l. of i.v. fluids more than controls in the first 5 postoperative days. In addition, net fluid balance on second postoperative day, in which POAF had the highest rate of incidence, was an independent predictor of POAF (OR 6.4, 95% CI 1.4 to 29.1, *P* = .014) [82]. 

In light of these results, maintaining a state of euvolemia is indicated to reduce the risk of POAF (see Figure 2). A judicious perioperative use of i.v. replacement fluids, with patient-targeted infusions and hemodynamic monitoring, would be necessary to achieve optimal tissue perfusion without overloading the heart and lungs [85, 86].

### 2.4. Hypoxia

Hypoxia has a causative role for perioperative supraventricular arrhythmias. Hypoxia leads to a reduced oxygen arterial content, that is, reduced oxygen delivery to tissues. Therefore, increased sympathetic activity aims to increase heart rate and enhance vascular tone to increase cardiac output and compensate for reduced arterial oxygenation. In postsurgical patients, hypoxia-related hyperactivity of sympathetic system may be responsible for development of POAF (see Figure 1). Acute hypoxia-driven pulmonary vasoconstriction can acutely overload right ventricle and cause right atrial overdistension and myocardial stretch. This may trigger POAF. Moreover, acute hypoxia is able to cause ischemic injury to atrial cells and myocardial conduction tissues, altering the electric properties of cells' membrane and triggering atrial arrhythmias (see Figure 1). Thus, POAF may be associated with postoperative respiratory failure, and all strategies aiming at the optimization of gas exchange can reduce the risk for POAF [87]. 

Chronic hypoxia, like in COPD or obesity, is a well-established risk factor for atrial fibrillation in the perioperative period. Pathophysiology of POAF in patients with chronic hypoxia includes polyglobulia and hypoxia-induced pulmonary vasoconstriction [88]. Increases in blood viscosity and pulmonary resistance induce an increase in right ventricular afterload, thus overloading right ventricle and atrium. This may lead to atrial cells' stretch and predispose to the electrophysiological alterations typical of POAF. Moreover, chronic hypercarbia increases atrial volume and pressure through sympathetic-mediated vasoconstriction [89].

Mooe and colleagues found that POAF had a significantly higher incidence in patients with obstructive sleep apnoea syndromes (OSASs) than in controls (39% versus 18%) [90]. Interestingly, multivariate analysis showed that predictors of AF recurrence were the magnitude of nocturnal oxygen desaturation and the proportion of time asleep with an oxygen saturation lower than 90%.

Kanagala et al. found that relapse of atrial fibrillation one year after electrical cardioversion was significantly and markedly higher in patients who had untreated OSAS than in patients who had been treated (82% versus 53%) [91]. In a retrospective study, Patel and et al. compared the incidence of postoperative complications in patients with OSAS. Incidence of POAF was greater in the OSAS group patients than the control group ones, and this value decreased strongly in all OSA patients treated with preoperative CPAP [92].

Optimization of oxygenation in patients with chronic respiratory failure would be associated with a reduced risk of POAF (see Figure 2). Possible strategies may include perioperative noninvasive ventilation, physiotherapy, or incentive spirometry [93]. In selected cases, preoperative hyperbaric oxygenation can be considered to reduce after CABG complications, including POAF [87].

### 2.5. Anemia

Perioperative anemia is a known risk factor of POAF. Anemia, especially acute anemia, produces an intense adrenergic activation and an increase in cardiac output finalized to increase cardiac output and compensate for reduced oxygen arterial, thus maintaining tissue oxygen delivery. The anemia-related adrenergic response may trigger POAF in predisposed patients (see Figure 1). Not uncommonly, atrial fibrillation may be one of the first signs of acute postoperative bleeding [94]. Another mechanism linking anemia to POAF is a relative ischemic injury to atrial myocytes and myocardial conduction cells, with altered membrane function and subsequent arrhythmic events (see Figure 1).

Several studies indicate therefore anemia as an important risk factor in the development of postoperative atrial fibrillation [18]. Despite that hemoglobin optimization is usually undertaken in the routine clinical care of surgical patients, there is not definitive evidence showing that increasing hemoglobin concentration up to a defined level necessarily improves outcome and reduces incidence of POAF. In cardiothoracic surgery, on the contrary, there is increasing evidence that packed red cell transfusion actually increases incidence of POAF [95]. In cardiothoracic patients, Sood and colleagues showed that patients who received postoperative transfusions had a 2-fold increase in their risk of developing POAF. According to the authors, the mechanism underlying this phenomenon may be related to a transfusion-related inflammatory response and to fluid overload [95]. Thus, despite that no definitive evidence exists, it seems reasonable that a minimum level of hemoglobin concentration should be set and achieved for each patients, based on clinical conditions and type of surgery, to optimize oxygen delivery (see Figure 2). However, overzealous use of blood products should be discouraged in view of associated adverse effects [96].

### 2.6. Hypothermia

Postoperative hypothermia has been associated with increased sympathetic activity and POAF (see Figure 1) [71]. In a prospective randomized study, Sun et al. demonstrated that the sympathetic response depends on the depth of hypothermia [97]. At lower temperatures, wider changes in plasma norepinephrine and neuropeptide Y were observed during the rewarming phase. Moreover, new-onset atrial fibrillation during the postoperative period occurred more often in patients who were actively cooled to 28°C (moderate hypothermic group) compared with those who were only cooled to 34°C (mild hypothermic group) (16.6% versus 66.7%, *P* = .03). Interestingly, recent guidelines on POAF prevention by the American College of Chest Physicians recommend the use of mild hypothermia for reducing the incidence of atrial fibrillation after cardiothoracic surgery [37]. It has been argued that mild hypothermia suppresses sympathetic nerve activity during rewarming following surgery, thus preventing neurohormonal mediated atrial fibrillation [98]. However, in noncardiothoracic surgery, maintaining normothermia is essential to prevent POAF and other cardiovascular complications (see Figure 2) [99].

### 2.7. Metabolic Alterations

Surgery-related metabolic alterations can trigger POAF in predisposed patients [18]. Marked metabolic acidosis, with consequent catecholamines secretion and myocardial sensitization, directly causes supraventricular and ventricular arrhythmias [94].

Perioperative hypoglycaemia can be associated with an increased susceptibility and longer duration of POAF (see Figure 1) [22]. In diabetic patients this effect is even more pronounced and hypoglycaemia should be considered as a potential reversible cause of atrial fibrillation in diabetic patients [100]. Actually, during hypoglycaemic episodes, the intense adrenergic stimulation is thought to cause arrhythmias [101]. 

Perioperative hyperglycaemia can also be detrimental for myocardial function, particularly after cardiothoracic surgery [102]. This is because elevated glucose concentration may induce damage to myocardial cells' membrane damage and alter its electrical properties, thus leading to POAF [103]. High blood glucose may generate free radicals and cause oxidative injury to myocardial cell, inducing apoptosis and, consequently, arrhythmias [104]. The myocardial oxidative injury can also be induced and maintained by hyperglycaemia-related secretion of cytokines, which enhances perioperative inflammatory (see above). Thus, even though strict glycemic control is still debated in the surgical and critically ill patient, avoiding excessive hyperglycaemia seems to be important to reduce cardiovascular complications after cardiothoracic surgery (see Figure 2) [105].

Hypothyroidism and subclinical hypothyroidism have adverse effects on the cardiovascular system and may cause cardiac arrhythmias and POAF (see Figure 1), particularly after CABG [106], and thyroid dysfunction is considered a predictor of poor prognosis for patients after surgery [107]. Pathogenesis of AF in patients with low levels of T3 hormone is not completely understood; a potential explanation was suggested from in vitro studies which showed that a low T3 state was associated to alterations of intracellular compartments for Ca2+ ion and generation of altered ionic flows across myocardial cell membrane [108].

It is well known that patients undergoing surgery, and especially cardiac procedure, have low free T3 levels due to decreased cardiac 5′-monodeiodinase activity [109]. This “low T3 syndrome” can be further worsened by preoperative hypothyroidism or preoperative subclinical hypothyroidism and is associated with POAF [106]. Furthermore, Klemperer et al., in a prospective randomized study, demonstrated that administration of exogenous T3 was able to reduce the incidence of AF in patients undergoing CABG [110]. 

Thus, detection of preoperative thyroid dysfunction could be important for patients undergoing surgery, particularly cardiothoracic, and preoperative thyroid hormone replacement therapy or supplementation would be beneficial in reducing incidence of POAF (see Figure 2) [106].

### 2.8. Electrolytes

In a retrospective survey, 23% of patients who developed supraventricular arrhythmia after noncardiac surgery had a metabolic derangement and an electrolytic imbalance [94], and a tight preoperative control of electrolyte imbalance is recommended to decrease incidence of POAF (see Figure 2) [111].

Potassium and magnesium deficiencies have all been associated with the development of atrial fibrillation in postoperative period (see Figure 1) [71].

In a prospective observational study, Walsh et al. [112] reported the incidence of and the risk factor for arrhythmias following noncardiothoracic surgery. Pre- and postoperative serum potassium levels were significantly lower in arrhythmia group than in control group (69% versus 24%; *P* < .005). These patients may have had intracellular potassium depletion at the time of the arrhythmia [113].

Magnesium deficiency may precipitate arrhythmias [114]. The use of magnesium in the preoperative and early postoperative periods is highly effective in reducing incidence of POAF after CABG [115], and magnesium can be considered as the antiarrhythmic drug of choice in depleted patients [11]. On the other hand, magnesium sulphate infusion does not decrease the rate of postoperative atrial fibrillation during the early postoperative period in normomagnesemic patients [116].

## 3. Conclusions

Postoperative atrial fibrillation is common and contributes to a worse outcome of surgical patient. Pathogenesis of POAF is multifactorial. The increased sympathetic outflow, related to hypovolemia, anemia, hypoxia, or pain, can elicit new-onset atrial fibrillation in surgical patients. Pericardial manipulation and local inflammation during thoracic and cardiac surgery may contribute, as does the surgery-related systemic inflammatory response. Finally, metabolic derangements such as perioperative hypothyroidism or hyperglycemia and electrolyte disturbances may also contribute to POAF pathogenesis. Thus, beyond pharmacological prophylaxis, several strategies can be implemented to reduce incidence of POAF, depending on which factor is involved. An adequate control of postoperative pain is mandatory, and the use of thoracic epidural analgesia for thoracic and upper abdominal surgery can induce a functional sympathetic block with negative chronotropic effect. The optimization of perioperative oxygen delivery, with a judicious and patient-tailored use of perioperative noninvasive ventilation and blood transfusions, may reduce the incidence of POAF. Perioperative fluid management should be targeted to avoid both hypo- and hypervolemia. Possibly, modulation of surgery-associated inflammatory response with immunonutrition and antioxidants may help reducing incidence of POAF. Perioperative depletion of potassium and magnesium should be corrected. Further research is mandatory to clarify the role of those interventions on incidence of POAF and outcome of surgical patients.
